# The Impact of Relational and Organizational–Environmental Aspects in Hospital Blood Collection: Clinical and Health Indications and New Training Needs

**DOI:** 10.3389/fpubh.2021.661530

**Published:** 2021-05-25

**Authors:** Antonio Iudici, Donata De Donà, Elena Faccio, Jessica Neri, Michele Rocelli, Gian Piero Turchi

**Affiliations:** ^1^Department of Philosophy, Sociology, Education and Applied Psychology, School of Human and Social Sciences and Cultural Heritage, University of Padua, Padua, Italy; ^2^Istituto di Psicoterapia Interazionista Psicopraxis, Padua, Italy

**Keywords:** patience perspective, blood collection, clinical implications, hospital management, healthcare service, health

## Abstract

This study deals with people who underwent a blood test and consequently suffered a fainting episode in the past. This phenomenon affects many people and if not adequately dealt with, it can lead to a perception of the blood test as a serious and traumatic event, which could limit its use as a preventive diagnostic tool. These experiences have been explored by research mainly on the basis of the physiological mechanisms involved in fainting, with a few studies considering the actual lived experience related to the blood test. This study explored how this experience is lived and managed, capturing aspects that could facilitate blood tests and the procedures associated with them, thus it focused on people with vasovagal syncope and was articulated through the semi-structured interview methodology. Among the significant results is the importance of the relational aspects implemented by health staff, the differing organisation of the blood test procedure, the need to make the hospital environment less aseptic and more humanistic, effective actions to counter the anxieties relating to the administration of the blood test and the importance of including the blood test with an inter-disciplinarity perspective.

## Introduction

Some people, under certain conditions, may experience episodes of transient loss of consciousness, T-LOC (also known as syncope), due to transient global cerebral hypoperfusion characterized by rapid onset, short duration, and spontaneous complete recovery (1).

When there is an emotional trigger (fright, strong emotion, pain, or unpleasant sensations of various kinds) or orthostatic (prolonged orthostatism), one may experience vasovagal syncope (VVS), which is a secondary reflex responsible for hypotension and bradycardia (2, 3), constituting about 30% of all syncope episodes (4, 5).

One of the most significant contexts in which vasovagal syncope occurs is the hospital environment, specifically in venous sampling situations. Such situations are particularly delicate, as the subject can faint, and if not adequately assisted, can fall to the ground unconscious, with possibly serious consequences (6). Fainting, experienced by a very large number of subjects undergoing sampling or vaccination procedures, generally occurs right after the procedure (7). The subjects involved exhibit some characteristic symptoms such as sweating, nausea, visual disturbances, tremors, sense of weakness and so on (pre-syncope or lipothymia) (8).

This can hence have a harmful effect on their health and may be capable of conditioning subsequent experiences in the healthcare sector. This is why the attention given to sampling becomes even more important. Indeed, in case of failure, there is the risk of being unable to obtain biological samples, fundamental for diagnosing any disease.

In epidemiological terms, VVS is a common phenomenon among the general population and the first episode occurs at a young age; it is experienced by about 1% of young children (9, 10). The prevalence of a first syncope episode is particularly high in patients aged 10–30, reaching ~47% in females and 31% in males around 15 years of age. In a cohort study, only 5% of the adult population had experienced a syncopal episode after 40, while the majority of young people and adolescents experienced a reflex syncope (1, 11).

Scientific studies available in the literature have devoted their attention primarily to the medical-physiological aspects of the problem (1), particularly focussing on understanding the mechanisms or the description of the causes of this phenomenon. Although the mechanism responsible for VVS has not yet been fully clarified, there is some data that throws light on part of the functioning: for example, a few studies on the afferent pathways and central processing (12). While the efferent pathways have been identified, arterial hypotension and bradycardia are related to a transient inhibition of the sympathetic system (13, 14) and an activation of the vagal system, respectively (15).

Other studies have paid greater attention to the psycho-physical aspects of fainting, exploring in depth the relationship between emotional and genetic aspects. Page (16) for example, has highlighted the importance of disgust and fear, as emotional aspects, in determining the psycho-physiological conditions necessary for the onset of vasovagal syncope. Other authors (17, 18) have focused on the reduction of syncope symptoms through specific techniques (applied tension technique), noting how the sense of disgust toward the needle would be strongly associated with needle phobia and would be virtually absent among patients who do not fear needles. Other authors have observed how the sense of disgust is linked to the idea of avoiding contact with and infection from contaminated items (19), where avoidance would produce increasing disgust. These recent studies have opened the door to the exploration of psychological factors involving the neuro-physiological activation (20), giving importance in particular to the identification of modalities for the reduction of symptoms and the ways in which the subject can perform actions that counter the fear of sampling and fainting (21, 22). The focus on the psychological aspects in recent literature prompted us to further investigate the subjects' lived experience of venous sampling or the experience of VVS, since studies focussing on this subject are missing in the literature. We have thus decided to explore this phenomenon with reference to the existential dimension of individuals who have concretely lived this experience. Hence, the present research has been performed starting from the interactionist perspective, which considers it relevant to investigate the meanings that subjects attribute to their personal experiences. We have decided to explore the phenomenon in a broad manner, identifying, however, some guiding questions derived from the literature review.

In particular, the research questions are as follows: What is the subjects' notion of a hospital, i.e., of the context in which fainting occurs? What idea do they have of the sampling, and how do they imagine it? How do they handle the sampling? Do they avoid it? What strategies do they adopt to reduce symptoms? How do people believe that a past fainting experience influences the present one?

## Methods

### The Participants

To answer the research questions, a cognitive survey was conducted on a sample of 73 subjects of both sexes diagnosed with pre-syncope or VVS (24 males and 49 females), with the age group 10–30, the average age being 19.06 (Table 1). The participants were subjected to blood sampling at the chemical-clinical analysis and microbiology laboratory of the Hospital of Belluno and Agordo. Participants consisted those who had manifested one or more episodes of lipothymia and/or fainting on that occasion or had reported having the experience in a similar situation previously.

**Table 1:** Summary of topic and questions.

Context configuration - How would you describe the context in which the blood sampling was taken, namely the hospital? - Describe which aspects could facilitate the blood sampling and which aspects could make it more difficult The configuration of the blood sample. - How would you describe the blood sample? - Describe what psychological and physical implications can be associated with the blood sample. - Do you think that blood collection is a method with specific (different) characteristics compared to other methods (e.g., X-rays, ultrasound or methods using needles such as vaccinations, allergic tests, etc.)? How to deal with the fear of withdrawal - Describe how you are dealing with the practice of blood sampling. - Do you adopt specific strategies to facilitate blood sampling? Which ones? (e.g., Do you talk to someone about it?) Role of previous experiences (fainting or discomfort). - How would you describe your past experience of fainting (or paleness, blurred vision, nausea, etc.)? - Describe the role that that experience plays in relation to the current blood sampling. - How do you deal with the blood sampling following that experience? Relationship between blood sampling and health. - How would you describe the blood sampling in relation to your health? - What are the specific effects of the blood sampling on your health?
Anagraphical variables: age, sex, body weight, fasting before collection, previous blood samples (number).

The following procedure was followed for the recruitment of the sample: initially, we obtained authorisation from the head of the Analysis Laboratory no. 1 to conduct and collaborate on this research. Thereafter, healthcare workers were appointed to select the sample. An informed consent form was submitted to the individuals so identified, requesting their permission to be contacted for the interview. On the day of the appointment, the participants received complete information about the research, the identity of the researchers and received answers to their queries, in accordance with the guidelines of the Helsinki Declaration, revised in 1989. The studies involving human participants were reviewed and approved by University of Padua Ethics Committee. The participants provided written informed consent to participate in this study.

### Objective

The research mainly focussed on the experiential dimension. Our efforts therefore emphasize on investigating the narratives of the participants. The goal was to explore how a subject with VVS experiences and manages blood sampling.

### Investigation Method: The Semi-structured Interview

Taking into account the exploration requirement found in the analysis of the literature, the investigation method was chosen to highlight the qualitative aspects of the investigated experience. For performing a wide but circumscribed survey of the sampling experience, the semi-structured interview was selected (23, 24). Another reason for this choice was the difficulty involved in gaining additional opportunities to interview people who undergo a sampling, as indicated by Bernard (25). The semi-structured interview is conducted following a “guideline” that provides a list of topics and issues to be discussed. It contains open questions of different kinds, prepared in advance by the researcher to maximise the acquisition of data and also considering the apprehension the subjects may experience during the interview. This way of conducting the interview is characterised by a low degree of control. In fact, the role played by the interviewer in “directing” and conducting the interview is limited. This method grants ample freedom to both the interviewee and the interviewer, while ensuring that all the issues identified are discussed, all the necessary information is collected, and that the specific perspectives can be expressed in the interviewees' own terms (26) (Table 2).

**Table 2:** Age and gender distribution of the study participants.

	**10–14 year**	**15–18 year**	**19–28 year**	**Tot**	**Average^a^**	**Std. deviation^a^**	**Std. error Mean^a^**	***P*-value^b^**
M	5	6	13	24	18.75	3,779	0.771	
% within gender	20.8%	25%	54.2%	100%				
% within Age/Bands	35.7%	31.6%	32.5%		32.9%			
F	9	13	27	49	18.82	4,563	0.652	
% within gender	18.4%	26.5%	55.1%	100%				
% within Age/Bands	64.3%	68.4%	67.5%		67.1%			
Tot.	14	19	40	73	18.79	4,295		0.951
% within gender	100%	100%	100%	100%				
% within Age/Bands	100%	100%	100%	100%				

a *Mean, standard deviation and standard errors refer to the age of the participants*.

b *P-value refers to the mean ages of men and women*.

### Data Collection and Analysis

The data was collected through a semi-structured interview starting from a grid of potential topics concerning the phenomenon in question. The interviews were recorded verbatim and subsequently transcribed for analysis. Some field notes were also been taken to capture non-verbal responses to questions. After collection and cataloguing, the responses were subjected to a synthesis activity for identifying the essential elements. Starting from these elements, initially unconnected to each other because they present a fragmentary way, we sought an interpretation that could establish new relationships between the elements, to enable new ways of understanding the phenomenon.

The categorisation and analysis were performed by two researchers independently, subsequently re-evaluating the non-congruent aspects (27). The analysis was conducted through the conceptual principles of the interactionist perspective (28–30).

## Results and Discussion

From the analysis, five macro response categories were identified, which have been indicated and described below. The identification of the categories was performed to grasp the most significant dimensions concerning venous sampling and the related issues.

### The Configuration of the Hospital as a Place Without Relationships

About half the people interviewed described the hospital as a generally favourable place: a safe, quiet, comfortable and clean environment. It is staffed by extremely knowledgeable and helpful operators who strive to deal with sickness in the best way possible. More than a third of the interviewees, however, described the hospital as a non-welcoming environment: not a nice place, detached and cold environment. These respondents described it as an ambience that instils fear and anxiety.

Among the aspects that could facilitate the sampling, many respondents indeed indicated the relational ones. These includes the following: the operator should speak to the patient, ask questions and distract them, use suitable and colloquial language, put the patient at ease, be helpful, kind, sympathetic and considerate. Some have specifically said they could be helped by “*a good relationship with the user*,” “*a relaxed atmosphere and a certain humanity on the part of the operators*” and in some cases, posture and contact are also important: “*shaking hands*.”

Conversely, some relational aspects were considered unhelpful for example, the operators' silence, cold, detached attitude, lack of sympathy for the uncomfortable situation experienced by the patient or when the procedure was rushed: “*To facilitate the sampling, staff should be kind, available and calm. While coldness and detachment toward what I feel would hinder the sampling.”*

According to the interviewees, another aspect that could facilitate the sampling concerned the organisation and the environment. Many explained the need to carry out the sampling in small environments “*since they give the sensation of greater warmth*” and to have relaxation tools available, for example “*having the opportunity to lie down on a bed or an armchair*.”

Among the organisational aspects considered dysfunctional, the waiting time was unanimously mentioned as a key stress-inducing factor. According to many, it instilled worry and fear, so much so that some reported having repeatedly considered leaving without going through with the sampling. Other significant responses concerned the environment, for example, a bare room creating a cold milieu and producing the sensation of being considered “*a number, an object*.” In some cases, not knowing the profession of the operators produced insecurity for the patients; for example, when the patient is treated by young people, they might be perceived as “*people with little experience or trainees*” and this fuels the concern.

### The Configuration of the Blood Sample as a Negative Event

Almost all the respondents considered sampling as an extremely negative event: a moment of tension and panic, scary, discomforting or invasive. Some regarded sampling as “*torture*,” “*one thing I have to do*,” “*a small traumatic event, even if easily overcome*.” Among the psychological implications, those particularly feared by the subjects were anxiety (half the sample), fear, and panic. The sampling is represented as a suction of blood from the veins. Physical implications include loss of consciousness (fainting), sense of fainting and other symptoms such as weakness, sweating, nausea, blurred vision, dizziness, discomfort, ear- buzzing, tremors and pain in the arm and stomach.

Many respondents were convinced that the physical aspect is far less important than the psychological one, which would have a decisive weight in the anxiety experienced regarding the sampling: “*it's all psychological, what you feel physically is definitely connected to the psychological aspect*.” Some described it as an altered state of consciousness, an experience that changes the sense of time and felt strange “*it's a short amount of time but seems a long time*.”

To a lesser extent, some respondents described the sampling as a quick procedure, but the significant aspect concerned viewing the sampling from a healthcare perspective, “*It's a quick action that's good, it's good for your health.”*

With reference to the comparison between blood sampling and other procedures, some subjects believed vaccination to be more harmless than sampling, because through the latter, “*blood is taken from you*,” i.e., they felt something being taken from their body. Others considered blood sampling to be different from vaccination, because of the different locations used by the two methods, that for sampling being the inside of the forearm, a part considered very delicate, unlike the area of the deltoid used for vaccination.

### Avoidance as an Elective Strategy

The participants responded by highlighting some strategy and actions, mainly at the behavioural level: “*I close my eyes or I avoid looking,”* “*I hope it goes well and is fast*.”

Some people requested the use of a needle meant for children. Others used a hot water bottle to dilate the veins. The answers seem related to an experience expected, felt to be strongly problematic and negative. Some of these actions relate to avoidance and are applied to thought, worry, fear and looking, and confirm the findings of other studies. These attempts often fail to achieve the desired effects, as anxiety and fear are actually exacerbated by them (31, 32).

Other solutions implemented involve physical actions, as if it were a chronic disease. Some patients, for example, seek to lie down to avoid falling in case of fainting, even if they have never fallen; or they ask to be accompanied by someone or ask for assistance, as if something irremediable could happen, for example being afraid of not waking up.

### Past Experience as the Cause of Present Problems

With reference to the role of past fainting experience, the subjects causally connected the current fear with an experience lived in the past “*in my opinion, these symptoms are due to the beginning of my fainting, when I have experienced some dizziness*.” Many respondents declared that nothing had changed since the first negative experience; they still viewed blood sampling in a very negative way.

It is as if people feel traumatised by their past experience and consider themselves victims of the same. The need that we notice is therefore for helping these patients to counter the causal logic underlying these experiences, which could lead to an excessive simplification of the experience and to relieve the subject of his or her responsibility with respect to the management strategies to be adopted.

### Sampling as a Tool of Health or as an Activity for Its Own Sake

Most of the subjects responded by describing the sampling as an important tool, which they could undergo to ascertain their state of health and detect possible problems in advance, “if I want to know, I have to suffer.” Some people reported undergoing the sampling when being sick, when it had been necessary; otherwise, they avoided it “it's something I have to do but knowing that I'm doing it to be better I'll do it willingly.” What emerges from the answers is that sampling is included in a discourse aimed at preserving health and is not considered independent of it.

In fact, when the sampling is clearly and explicitly linked to health interests, the interviewees' narratives reflect reasoning by objectives. In these cases, the sampling is considered useful, functional or aimed at acquiring data on one's own health.

Other patients view withdrawal as an end in itself, separate from health. In the latter case, there is a greater judgment regarding the sampling and the activation of behaviours to avoid suffering.

This shows the need to help these patients to relate the experience of the sampling in itself, making it part of the narrative, in which the subject explicitly reasons in terms of responsibility toward his or her own health.

## Discussion

The interviews allowed us to highlight the way the participants configured some fundamental aspects of this experience, leading to some reflections that could be translated into guidelines for improving the support for people suffering from these disabling concerns.

The answers showed that most respondents considered the hospital a safe place, but this idea did not decrease the concerns regarding the sampling, which was still considered an extremely negative event, as we shall see below. What respondents considered crucial in reducing or increasing the fear of sampling and fainting were the relational and organisational-environmental aspects implemented by the hospital staff.

This suggests a specific consideration regarding the importance of the personnel involved in the sampling, and not necessarily solely the person who directly carries out the procedure (33). In fact, the venous sampling procedure is generally carried out in a routine manner and with a work organisation which aims at reducing the time necessary for each operation. In this context, therefore, the possibility of a prolonged comforting interaction with the patients is necessarily limited.

However, within the aforesaid constraints, even a simple word or a gesture of attention and care toward the person can be an important help (34, 35). This is further confirmed by the interpretation of the patients regarding the actions of the operators: indeed, the routine attitude, which often signifies a formal, aseptic and detached approach, is perceived as a sign of non-attention and lack of consideration for patients. In this sense, there is a need to train specialised personnel for a brief communication exchange with these patients, to secure greater cooperation and to afford them a better perception of well-being and control (36).

On the other hand, as regards organisational aspects, the excessive waiting before the sampling was found to be a key aspect negatively affecting the sampling experience. Indeed, the concern increases in direct proportion with the duration of waiting, according to the majority of respondents.

Sampling is configured as a source of anxiety and tension, mainly due to the fear linked to the needle, and often experienced as a trauma. The needle used in the course of the venous sampling is often perceived by the subjects as menacing, an invasive instrument that insinuates itself into the body, “picking up” an element of the person, and therefore as a factor capable of threatening the identity of the subject. More specifically, respondents mentioned the characteristic symptoms of lipothymia or pre-syncope, which, for many of the interviewed subjects, would be strongly affected also by their mental representations prior to the sampling. It would therefore be precisely the way in which the subjects represent to themselves the event of the sampling and the connected fear of the needle to facilitate those physical implications which, in certain situations, can contribute to the onset of the psycho-physical mechanism that characterises lipothymia and which began before the event itself; for some of them, even a few days before.

In fact, from the research data, we can make the following two observations:

(1) For the interviewed subjects, the psychological implications of anxiety, fear and discomfort originate always before venous sampling, and never after; (2) It is probable that such emotional manifestations (anxiety, fear, discomfort) are strongly affected by the way in which the subject imagines the future event of the venous sampling.

In these latter cases, therefore, considering the significant temporal distance that separates the current anxious emotional state from the future venous sampling, it is possible to conclude that this emotional state is mostly the result of how the sample is configured in the anticipation. The considerations just carried out therefore allow us to hypothesise that the configuration of the venous sampling, elaborated by these subjects at a time before the sampling itself, constitutes an important pre-condition for the physical and psychological implications reported by the subjects. We should indeed consider that the mechanism of lipothymia develops in a relatively short time, placed astride the sampling itself. Almost all subjects faint invariably after the venous sampling, but anxiety, fear and discomfort begin to manifest shortly before the event.

A limitation of this research work can be the age range of the participants involved, which covers youth and minors, as well as young adults. It might be useful to see if there are other specific differences between these two groups, and if there are any other differences between the middle-aged and elderly population. Another limitation concerns the gender of the participants. Females were about twice the number of males. Although the standard deviation did not show significant aspects in terms of gender, it might be useful to investigate the male gender more critically.

## Conclusions

This research first highlighted the importance of healthcare staff–patient interactions, as they can impact patient care and health. Consequently, there appears to be a clear need to train employees to adopt a welcoming attitude and be communicative with patients. This can greatly facilitate both collaboration and improving the hospital experience, as also found by Hughes et al. (37).

A second significant element concerns organizational aspects, which show that fear and anxiety grow with the patient's perceived wait duration, leading to arrival at the withdrawal procedure with anticipations that strongly increase their tension and fear. Sibbald and Kothari (38) and Bijttebier and Vertommen (39) support this aspect.

This demonstrates the need to better prepare the retrieval locations and manage the waiting time to make it less anxiogenic. It might be helpful to research international setups that can reduce fear.

Most of the strategies used by the respondents were behavioural interventions based on avoidance, such as trying not to think, not to worry, etc. These strategies are counter-productive, because the effect is increased thinking. Due to this paradoxical effect, it would be advisable to organise courses for patients on the management of anxiety that go beyond mere behavioural control (40).

Past fainting experiences were considered by the interviewed patients as causally responsible for the current concern, leading to the subject considering himself or herself dominated by their personal past.

To conclude, it is necessary to help patients in terms of causality and manage their health responsibly and actively (41–44). Research also shows that patients who explicitly viewed sampling as instrumental to their health had a better experience. Consequently, it becomes important to train clinicians to place sampling within health-based discourses and not as a stand-alone tool.

## Data Availability Statement

The raw data supporting the conclusions of this article will be made available by the authors, without undue reservation.

## Ethics Statement

The studies involving human participants were reviewed and approved by University of Padua Ethics Committee. The participants provided written informed consent to participate in this study.

## Author Contributions

DD and AI: conceptualization and writing—review and editing. AI, DD, and EF: methodology. DD, AI, and JN: formal analysis and investigation. DD: writing—original draft preparation. JN and MR: resources. EF and GT: supervision. All authors contributed to the article and approved the submitted version.

## Conflict of Interest

The authors declare that the research was conducted in the absence of any commercial or financial relationships that could be construed as a potential conflict of interest.
